# The strengths and limitations of routine staging before treatment with abdominal CT in colorectal cancer

**DOI:** 10.1186/1471-2407-11-433

**Published:** 2011-10-07

**Authors:** Irene Grossmann, Joost M Klaase, Johannes KA Avenarius, Ignace HJT de Hingh, Walter JB Mastboom, Theo Wiggers

**Affiliations:** 1 Department of Surgery, University Medical Center Groningen, (Hanzeplein 1), Groningen, (9713 GZ), the Netherlands; 2 Department of Surgery, Medical Spectrum Twente, (Haaksbergerstraat 55), Enschede (7513 ER), the Netherlands; 3 Department of Radiology, Medical Spectrum Twente, (Haaksbergerstraat 55), Enschede, (7513 ER), the Netherlands; 4 Department of Surgery, Catharina Hospital, (Michelangelolaan 2), Eindhoven, (5623 EJ), the Netherlands

## Abstract

**Background:**

Advanced colorectal cancer (CRC), either locally advanced, metastasized (mCRC) or both, is present in a relevant proportion of patients. The chances on curation of advanced CRC are continuously improving with modern multi-modality treatment options. For incurable CRC the focus lies on palliation of symptoms, which is not necessarily a resection of the primary tumor. Both situations motivate adequate staging before treatment in CRC. This prospective observational study evaluates the outcomes after the introduction of routine staging with abdominal CT before treatment.

**Methods:**

In a prospective observational study of 612 consecutive patients (2007-2009), the ability of abdominal CT to find liver metastases (LM), peritoneal carcinomatosis (PC) and T4 stage in colon cancer (CC) was analysed.

**Results:**

Advanced CRC was present in 58% of patients, mCRC in 31%. The ability to find LM was excellent (99%), cT4 stage CC good (86%) and PC poor (33%). In the group of surgical patients with emergency presentations, the incidences of both mCRC (51%) and locally advanced colon cancer (LACC) (69%) were higher than in the elective group (20% and 26% respectively). Staging tended to be omitted more often in the emergency group (35% versus 12% in elective surgery).

**Conclusions:**

The strengths of staging with abdominal CT are to find LM and LACC, however it fails in diagnosing PC. On grounds of the incidence of advanced CRC, staging is warranted in patients with emergency presentations as well.

## Background

Advanced colorectal carcinoma (CRC), defined as locally advanced or metastasized disease or both, is present in a relevant proportion of patients diagnosed with colorectal cancer. Common localizations for distant metastases are the liver, the peritoneal cavity and the lung. Staging with chest CT as a routine procedure before surgery has not shown to be of clinical benefit, mainly due to the low incidence of clinically relevant lung metastases and low specificity of chest CT [1-3]. Pre-operative staging with abdominal CT might be beneficial when the ability to detect advanced CRC is high enough and the findings offer information that may change the treatment plan. Such findings include liver metastases (LM), peritoneal carcinomatosis (PC) and locally advanced colon cancer (LACC). In the past, these conditions were frequently regarded as incurable and suitable for palliative measures only. Nowadays various multi-modality treatments offer a chance of cure to selected patients [4-15]. For patients with incurable advanced CRC, alternative options are sometimes available and staging may change the treatment plan towards the 'best palliative care', that may consist of avoidance of surgery in selected patients [16-18]. At the present, adequate staging with CT before treatment is already considered as the standard in international CRC guidelines. However the evidence for this advice in literature is mostly limited to studies focused on the radiological accuracy and also are usually conducted in selected patient groups. The actual findings of staging after implementation of such guideline has not been described before. An observational study may be helpful in the debate on actual clinical relevance and provide lead points to optimize staging in clinical practice.

The aim of this study is to describe the outcomes of routine staging with abdominal CT in a unselected hospital population (n = 612) after the introduction of a regional guideline recommendation in 2007.

## Methods

The data were collected in the Medical Spectrum Twente, a large community teaching hospital in the regional capital of a foremost rural area in the eastern part of the Netherlands. It functions as a regional referral center for liver and lung surgery, but has no facilities for the treatment of peritoneal carcinomatosis with hyperthermic intraperitoneal chemotherapy (HIPEC).

The study design is a prospective observational cohort study evaluating the outcome of routine staging with abdominal CT concerning the ability to find liver metastases (LM), peritoneal carcinomatosis (PC) and T4-stage in colon cancer (LACC). *All *patients in our hospital who where treated for CRC from January 2007 till December 2009 were included in the analysis; all surgical patients with CRC in the study hospital were prospectively registered in a database designed for colorectal surgery, including patient characteristics, staging and surgical procedures, the clinical M stage and pathological TNM stage, post-operative mortality, treatment of metastases and follow-up; patients with the diagnosis of CRC in the same 3 years who did not undergo surgery were identified by the regional cancer registry and retrospectively added to the database. The clinical T stage of colon cancer on abdominal CT (cT4 or non-cT4) was retrospectively scored based upon the original radiology reports.

Routine pre-operative staging with a CT of chest and abdomen for patients with CRC was introduced as a regional CRC guideline in 2007 and preceded similar national guideline recommendations (2008). CT scanning was performed on a 16 and 64 slice scanner (Toshiba Aquillion 16 and 64) after intravenous contrast injection (visipaque 320, 90 ml, 3 ml/s.) in the portal venous phase, with a slice thickness of 1 mm and a reconstruction of 0.8 mm. When preoperative scanning was omitted, staging with abdominal CT was intended within 3 months after surgery. Patients with rectal cancer, defined as localization below the peritoneal reflection, were additionally staged with a pelvic MRI for determination of the local invasion and possible lymph node metastases (cTN stage) and received neo-adjuvant (chemo)radiation according to the Dutch guidelines on rectal cancer. Follow-up after curative treatment of non-metastatic CRC consisted of serum CEA measurements every 3 months combined with bi-annual ultrasound of the liver.

Pathological staging was based upon the TNM classification 2002 (6th edition) and classified according to the American Joint Committee on Cancer (AJCC) stages. Advanced CRC was defined as either locally advanced disease, presence of distant metastases or both. Locally advanced colon cancer (LACC) was defined by pT4 stage; meaning the tumor showed invasion through the serosal layer *or *into surrounding organs. Locally advanced rectal cancer (LARC) was defined as all patients that had either a T4 tumor or a T3 tumor with a threatened circumferential margin on pelvic MRI. The final diagnosis of liver metastases was based upon radiological (CT, contrast-enhanced ultrasound and/or PET scanning) and per-operative findings. In case of resection or in case of persistent uncertainty, histological confirmation was obtained. The final diagnosis of peritoneal carcinomatosis was by histological confirmation. The final diagnosis of lung lesions was on radiological grounds (chest CT). In case of resection or indeterminate lung lesions, histological confirmation was obtained when feasible. Several lung lesions remained indeterminate: these were followed by repeat CT scanning and considered positive when growth was observed [3]. Incurable CRC was defined as all macroscopical irradical (R2) resections of the primary tumor, when the patient had no resection of the primary tumor, or when no intended curative treatment of distant metastases was done.

Emergency presentation in the surgical patients was defined as all non-planned admissions to the hospital due to symptoms related to the tumor, with a subdivision into 'urgent' defined as surgery imperative within 5 days and 'acute' procedures within 6 hours.

The ability of the staging abdominal CT to detect advanced disease was analyzed in surgical patients that were staged with CT before treatment; the gold standard for PC and LACC were per-operative findings confirmed with histology. For LM the findings on CT (negative for liver metastases or indeterminate lesions) were related to per-operative findings and follow-up.

## Results

In a 3 year period (2007-2009), 612 patients were diagnosed with colorectal carcinoma in the study center. Staging with abdominal CT before treatment was done in 513 patients (84%). Surgery, either palliative or curative, was performed in 551 patients (90%). The proportion of surgical patients with an emergency presentation was 21% (115/551).

### Population incidences of advanced colorectal cancer (Figure 1 and Table 1)

**Figure 1:**
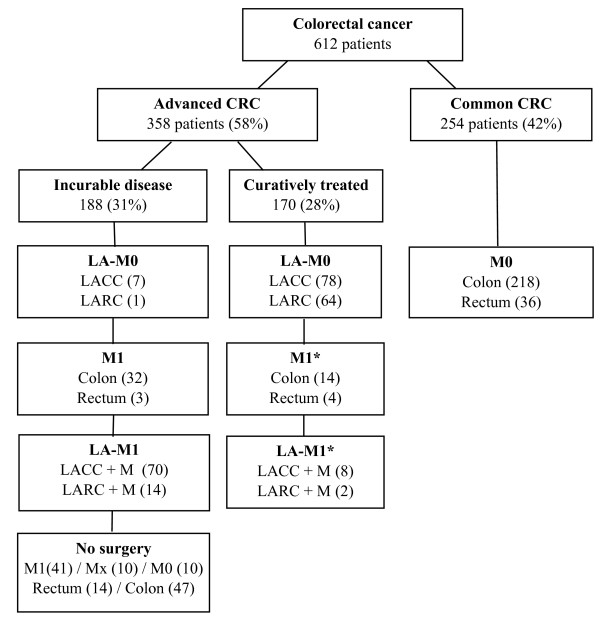
**Colorectal cancer stage and treatment entire cohort**. LA: locally advanced tumors (clinical on staging and/or pathological). M1: metastastic CRC. M0: no regional or distant metastases. LACC: locally advanced colon carcinoma = pT4 according to TNM 2002 (6th edition). LARC: locally advanced rectum carcinoma = T4 tumors and T3 tumor with threatened circumferential margin on MRI. *Curative treatment distant metastases in 28 patients: liver 24, peritoneal 2, lung 2.

**Table 1:** Characteristics all patients with CRC 2007-2009

	All patients (n = 612)	
*Age*		
Mean	70 yr	
Median	70 yr	
Range	33-98 yr	

*Gender*		
Male	349	57%
Female	263	43%

*Localization primary tumor*		
Colon	474	77%
Rectum	138	23%

*Staging procedure*		
Abdominal CT preceding treatment	513	84%
Abdominal CT < 3 months after surgery	44	7%

*AJCC stage based on pTNM (2002)^a^*		
Stage 0	12	2%
Stage I	75	12%
Stage II	171	28%
Stage III	143	23%
Stage IV	188	31%
Not classified^d^	23	4%

*Localizations of distant metastases in cohort^b^*		
Liver	101	17%
Peritoneal	27	4%
Liver and lung	24	4%
Liver and peritoneal	11	2%
Lung	7	1%
Peritoneal and lung	4	1%
More than 2 organs and/or other localisations^c^	14	2%

*Localization of distant metastases per organ^b, c^*		
Liver	144	24%
Peritoneal	49	8%
Lung	42	7%

^a ^This includes patients with pathological downstaging after neoadjuvant chemoradiation (n = 69); a complete remission of histologically proven colorectal cancer was staged as stage 0.

^b ^Staging of the lung in this cohort was done with chest CT (n = 415) or chest X-ray (n = 197).

^c ^Other localisations were brain and skeletal metastases.

^d ^Of 23 patients, 20 patients had no surgery and 3 patients had no resection resulting in an unknown TN status. Of 23 patients, 11 patients had an unknown M status and 12 patients had M0 status.

Metastatic CRC (mCRC) was diagnosed in 188 patients (31%). Most common sites of distant metastases were the liver (n = 144, 24%), peritoneal cavity (n = 49, 8%) and the lung (n = 42, 7%). Intended curative treatment of metastases was feasible in 15% of patients with mCRC (n = 28). Locally advanced disease was present in 262 patients (43%); on the total CRC population the incidence of LARC was 13% (n = 81) and of LACC was 30% (n = 181). Incurable disease was present in 188 patients (31%), mainly due to mCRC (n = 160).

### Finding mCRC and/or LACC on abdominal CT (Table 2)

**Table 2:** Detection advanced CRC on abdominal CT

*Analysis of surgical patients that were staged before treatment (n = 463)*								
			**Diagnosed**		**Suspected**		**Not seen**	

Liver metastases	n = 86		73	85%	12	14%	1	1%

Peritoneal carcinomatosis	n = 33		11	33%	6	18%	16	48%

pT4 colon carcinoma^a^	n = 115		99	86%	5	4%	11	10%

	**Rectum (n = 115)**				**Colon (n = 348)**			

	**cM**		**pM**		**cM**		**pM**	

	n	%	n	%	n	%	n	%

Liver metastases	13	11%	16	14%	61	18%	70	20%

Peritoneal carcinomatosis	2	2%	3	3%	9	3%	30	9%

Locally advanced disease	n.a.^b^		n.a.		99	29%	123	36%

cM: metastases diagnosed before treatment on staging abdominal CT (without additional diagnostics)

pM: final conclusion, including additional imaging, peroperative findings and histology

^a ^In 8 patients the local invasiness was not described in the original report, leaving 115 patients for evaluation

^b ^Local invasiveness and lymph node status (cTN stage) in rectal carcinoma was determined on pelvic MRI

The cohort of surgical patients that were staged before treatment (n = 463) was analyzed on the ability of the abdominal CT to find advanced disease. In this group, 86 patients had liver metastases; in 73 patients (85%) diagnosed and in 19 patients (14%) suspected (indeterminate lesions) on the initial staging CT. The indeterminate liver lesions were further evaluated with additional diagnostic testing, which were contrast-enhanced ultrasound, positron emission tomography (PET), PET/CT or MRI. In 12 patients the indeterminate lesions were diagnosed as metastases and in 7 patients as benign lesions; of these, 6 patients had a follow-up of more then one year including imaging of the liver, and none was diagnosed with metastases. In one patient the liver metastasis was not seen nor suspected on CT, but found during surgery. The ability of the staging CT to detect liver metastases, summating diagnosis on CT and with additional imaging for indeterminate lesions, was 99%. Peritoneal carcinomatosis was diagnosed in 33 patients; in 11 patients prior to surgery on the staging CT (33%), in the remaining 22 patients these were found during surgery for the primary tumor. LACC was correctly suggested in 99 out of 115 patients (86%).

In surgical patients with LACC (n = 160), the incidence of mCRC was 45% (n = 73); PC was present in 38 patients (24%) and LM in 46 patients (29%). Of the 41 surgical patients with PC, 38 patients had LACC (93%).

### Emergency presentation in surgical patients

In surgical patients with an emergency presentation (n = 115), the incidence of mCRC was 51% (n = 59) versus 20% in the elective group (88/436). In the same group, limited to colon cancer patients (n = 110), the incidence of LACC was 69% (n = 76) versus 26% in the elective group (84 out of 317). Staging was omitted in 35 out of 115 patients with an emergency presentation (30%), versus 53 out of 436 elective surgical patients (12%).

## Discussion and Conclusions

Advanced CRC is common, occurring in 58% of CRC patients in the study. It raises little question at the present that staging before treatment is required. It is presumed the abdominal CT can accurately identify the patients with advanced CRC. For liver metastases and LACC the abdominal CT indeed is an adequate first-line imaging technique, however for PC it grossly lacks sensitivity. Appreciating the relationship between LACC and PC, LACC may serve as a warning sign for the possible presence of PC. The incidence of advanced CRC was especially high in patients with emergency presentations, while staging tended to be omitted more often in this group.

The present study was done in a single hospital and therefore has a limited number of patients as compared to true population-based studies. The additive value of this analyses is the prospective design, that is inherently more accurate and provides more detailed clinical data. The outcomes are expected to represent population based incidences fairly good, due to the fact the study was done in an relatively isolated region in the Netherlands that knows very little referral or selection bias. In comparison to a large population-based study from the Netherlands [19] this study reports a higher incidence of mCRC (colon cancer 34% vs 25% and rectal cancer 22% vs 24%). This variance in incidence of mCRC probably has several causes; such as due to differences in staging routines, mode of data registration, CT scanning protocols and the adverse regional socio-economic status.

Considering potentially curable mCRC, the relevance and outcomes of staging before treatment is most well-known for liver metastases. Staging with CT before treatment provides the opportunity to change the treatment strategy; various studies have reported promising results of alternative approaches in terms of eligibility of resection and the oncological outcome [10,11,20]. Previous reports show a high accuracy of CT for liver metastases, with an initial sensitivity of approximately 85% which is similar in this study [21]. One study concluded that also small and indeterminate liver lesions should be reported, to optimize the detection rates of true metastases[22]. Our observations agree with that conclusion; Indeterminate lesions in the liver were not a major concern and discrimination of indeterminate liver lesions with additional imaging caused no major diagnostic uncertainties or resources. Contrast-enhanced abdominal MRI is an equivalent alternative to multi-slice CT scanning for LM, [23] however may cause more logistic problems.

Concerning PC, the benefits of staging before treatment are much less outspoken. In the recent past PC was regarded as a virtually incurable condition with little treatment options. Therefore accurate staging of PC was considered less important. This has changed since the introduction of HIPEC offering a chance for cure in selected patients [6,12,13]. Since HIPEC is performed in specialized centers only, accurate pre-operative staging of PC has become vital to improve the outcome of these patients. The present study confirms the finding of previous studies that PC is poorly visualized on a CT scan [24]. Other imaging techniques may be more sensitive to detect and estimate the extent of PC, such as diffusion-weighted imaging (DWI) combined with MRI [25]. This however remains to be proven for its clinical value in colorectal cancer. An alternate approach may be to utilize the observed close relationship between PC and LACC, e.g. by performing a diagnostic laparoscopy in patients with a cT4 tumor on CT; estimation of T stage on abdominal CT in this and another study [26] seems to be fairly reliable.

Standardized radiology reports on T stage in colon cancer and on localization, sizes, spread and preferably 'weighed' suspicion of distant metastases may enhance the ability to find advanced CRC with the staging abdominal CT.

## Competing interests

The authors declare that they have no competing interests.

## Authors approval

All authors have approved on the final version of this manuscript

## Authors' contributions

TW, IG and JK were responsible for the study design with TW as the principal investigator. IG, JK and WB were responsible for the clinical implementation of staging before treatment with CT, data acquisition and critical review of the manuscript. JA was responsible for the clinical implementation of staging with the abdominal CT at the radiology department, designing the actual scanning protocol, and he functioned as consultant on findings and (additional) imaging techniques. IdH was involved as expert on peritoneal carcinomatosis as co-writer of the manuscript, and functioned as critical reviewer. IG was the study coordinator (at the time of the study she worked in the Medical Spectrum Twente as a resident and the UMCG as a researcher) and wrote the manuscript.

## Authors information

IG: Surgeon, fellow in oncological gastrointestinal surgery. From february 2010 till july 2011 she worked as a fellow at the Catharina Hospital and starting from July 2011 she continues her fellowship at the Medical Spectrum Twente (MST). Further she holds a position as researcher at the UMCG. She previously (also) worked at the MST as a resident in general surgery. This study was part of her thesis "Searching for metastases in colorectal cancer" (University of Groningen).

JK: Oncological gastrointestinal surgeon, specialized in gastrointestinal and hepatobiliary surgery. Tutor for scientific research at the Medical Spectrum Twente.

JA: Staff radiologist at the Medical Spectrum Twente

IdH: Oncological gastrointestinal surgeon, specialized in treatment of peritoneal carcinomatosis (PC) and head of the research group on PC at the Catharina Hospital.

WM: Oncological and gastrointestinal surgeon, specialized in colorectal and head/neck surgery. Responsible tutor of the trainees in general surgery at the Medical Spectrum Twente.

TW: Professor in oncological surgery and former head of department at the UMC Groningen.

## Pre-publication history

The pre-publication history for this paper can be accessed here:

http://www.biomedcentral.com/1471-2407/11/433/prepub
